# Reducing inappropriate inpatient blood glucose monitoring

**DOI:** 10.1016/j.clinme.2025.100537

**Published:** 2025-11-29

**Authors:** Sophia Shuen Yii Ng, Yan Ling Lai, Pei Yi Lee, Karen Cera, Esther Melissa Michael, Xiao Juan Chen, Esther Huiyun Lin, Schneider Wong, Ya Yuan Nicole Chong, Desmond B Teo

**Affiliations:** Alexandra Hospital, National University Healthcare System, 378 Alexandra Rd, Singapore 159964, Singapore

**Keywords:** Blood glucose monitoring, Quality improvement, Inpatient, Clinical guidelines, Healthcare resources, Medical education

## Abstract

Inpatient blood glucose monitoring (BGM) is prevalent, while healthcare costs and workforce demands are rising. We aimed to identify causes of unnecessary monitoring and assess effectiveness of targeted interventions, to maximise healthcare resources while prioritising patient safety.

Clinical guidelines were established with the Endocrinology division. A root-cause analysis revealed different factors, which were targeted through the plan, do, study, act methodology. Education and raising awareness were provided to staff. Guidelines were summarised into emails and stickers. Data were collected after each intervention, to monitor inappropriate monitoring rates and adverse events (hypoglycaemia and hyperglycaemia).

Results revealed a reduction of inappropriate BGM from 49.5% to 9.2% over 7 months, with no significant change in adverse events, with estimated annual savings of S$564,818.80 (£421,185.38) and 663.4 nursing hours hospital-wide. This highlights the effectiveness of clear guidelines, targeted multidisciplinary education to bridge gaps and empower staff to collaborate for patient-centred, high-value care.

## Introduction

Blood glucose monitoring (BGM) is a prevalent component of inpatient care. However, unnecessary BGM leads to increased healthcare cost, inefficient use of nursing time and patient discomfort, without contributing to high value care.

In Singapore, the majority of BGM requests are initiated by junior physicians. Requesting a battery of tests is common but no longer sustainable, given limited healthcare resources. There are large practice variations in reviewing indications for the continuation and frequency of BGM. In the context of a heavy patient workload, time pressure, limited experience of doctors and differing practices, the indication for BGM may not be reviewed regularly. Clinicians attributed unnecessary ordering of laboratory tests to the health system culture, and lack of cost transparency and role models that demonstrate restraint.^1^^,^^2^ In Alexandra Hospital (AH), it takes approximately 1 min to obtain a glucose point-of-care test (POCT) for a patient, while the cost is S$14.19 (£8.19).

This is a problem prevalent beyond Singapore. In the UK, one in six hospital beds is occupied by someone with diabetes and this is predicted to rise to one in four by 2030.^3^ Rising healthcare costs and workforce shortage^4^ in the healthcare industry are also global challenges. A quality improvement project conducted in Norfolk found that 70% of BGM was non-compliant with guidelines. Education of staff reduced monitoring by 51.9%, with savings of 7.5 min of healthcare worker time and £0.75 in consumables per patient per inpatient day.^5^ Evidence-based clinical guidance has also been shown to reduce healthcare costs.^6^

The aim of our project was to identify factors contributing to high rates of inappropriate BGM, focusing on individual and multidisciplinary factors between different healthcare professionals. We believed that empowering doctors and collaborating with nurses would lead to mindful BGM. We aimed to reduce inappropriate BGM from 49.5% to 12%. We also monitored adverse events of hypoglycaemia and hyperglycaemia to ensure that patient safety was prioritised.

## Methods

This project was performed in a general medicine (GM) ward in AH. The team comprised one senior doctor, three doctors, and three nurses representing different priorities of healthcare roles.

**Fig. 1:**
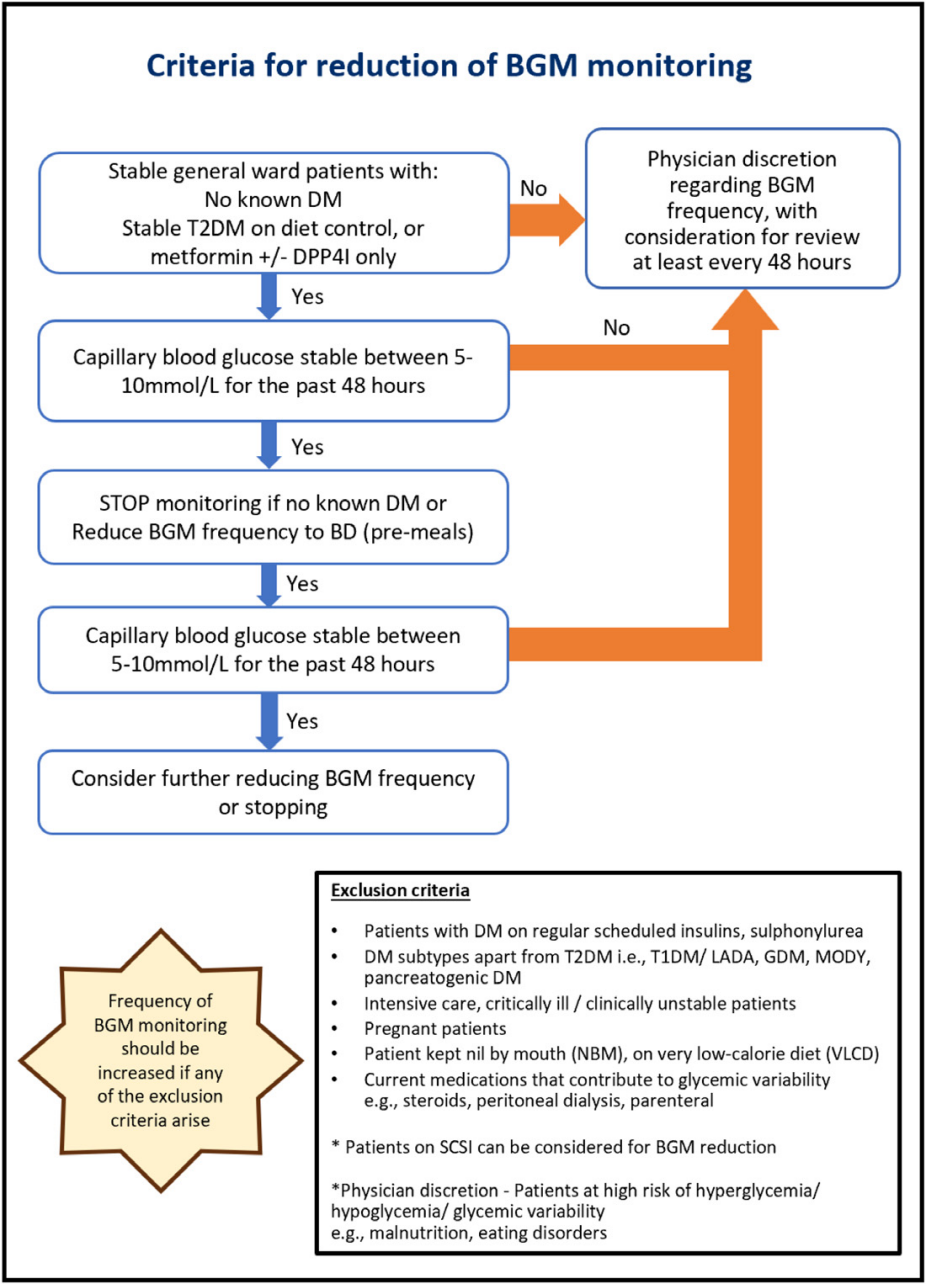
Established clinical guidelines for reducing inappropriate BGM.

We collaborated with the Endocrinology division to establish criteria for suitable patients, and a set of clinical guidelines to reduce BGM, adapted from the Endocrine Society Clinical Practice Guidelines,^7^ considering the side effects of ceasing BGM, varying complexities of admissions, concomitant procedures and/or medical/surgical issues (Fig. 1).

**Fig. 2:**
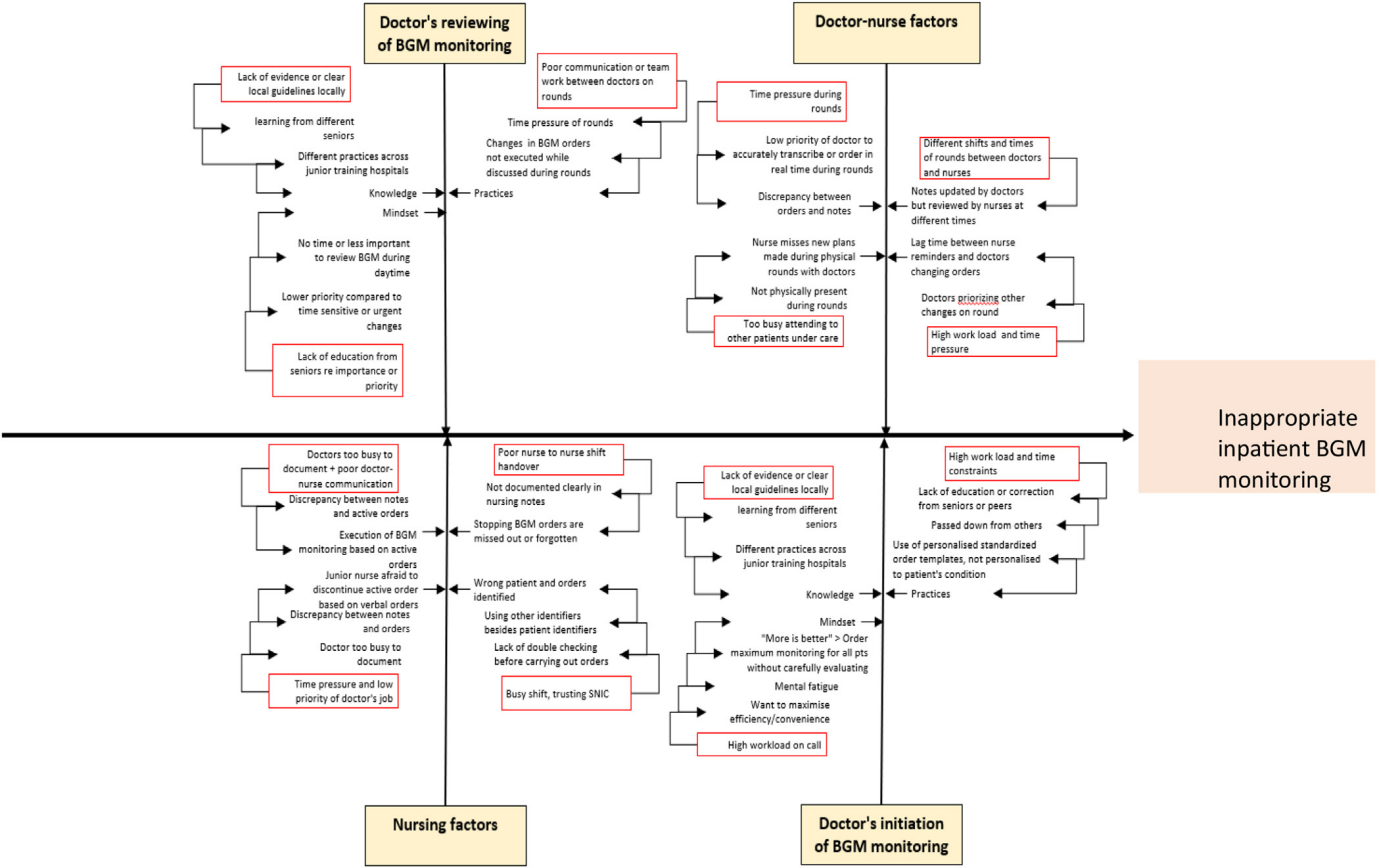
Fish bone diagram showing root cause analysis of inappropriate BGM.

**Fig. 3:**
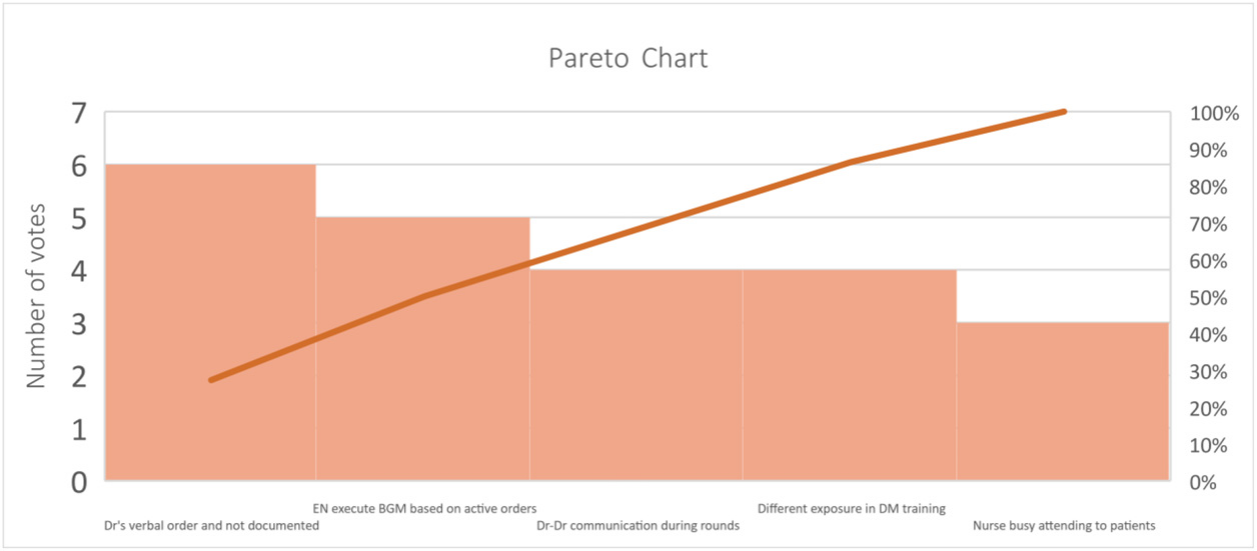
Pareto chart displaying the most significant factors contributing to inappropriate BGM.

A root-cause analysis was performed to identify factors contributing to inappropriate BGM (Fig. 2, Fig. 3).

We employed the plan, do, study, act (PDSA) methodology to target the root causes by:

1) Educating junior doctors who are posted to AH regularly to fill existing knowledge gaps and encourage a high-value care mindset.
2) Educating senior doctors through raising awareness of the project and guidelines during department meetings – ensuring that strong leadership and practices were aligned with positive role-modelling.
3) Educating nurses with emphasis on clinical guidelines during roll call – encouraging nurses to take proactive roles in nurse-led care and foster confidence to discuss cases with doctors.
4) Email reminders summarising clinical guidelines were sent to incoming staff before their ward cover.
5) Sticker reminders summarising clinical guidelines in an easy-to-read flowchart were printed and pasted onto computers-on-wheels (COWs) which doctors and nurses utilise to review patients daily.

BGM was performed by nurses according to doctors’ orders in the system as per usual practice. BGM appropriateness was audited by three junior doctors who screened a group of selected patients based on criteria and clinical guidelines. Data were reviewed from BGM and drug charts, clinical notes, and patients’ conditions in the hospital’s electronic record system.

Adverse event rates, hypoglycaemia and hyperglycaemia were measured prior to initiation of project followed by using de-identified data from the hospital’s Value Driven Outcome Office.

**Fig. 4:**
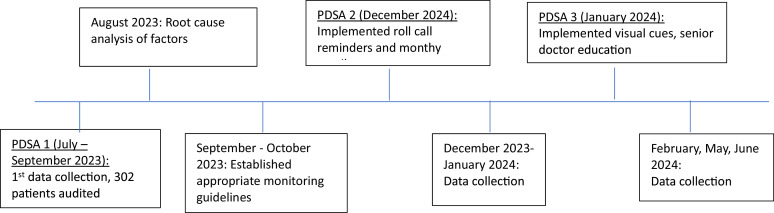
Timeline of project.

Timeline of project (Fig. 4).

### PDSA cycle 1: baseline data collection

A total of 302 patients from July to September 2023 were audited to acquire baseline data on the frequency of inappropriate BGM before interventions’ implementation.

### PDSA cycle 2: implementation of clinical guidelines with roll call reminders, monthly emails and education

This cycle aimed to evaluate the change in behaviour of healthcare staff after roll call reminders, monthly email and education sessions.

### PDSA cycle 3: implementation of senior doctors education and physical sticker reminders

Qualitative feedback was collected between PDSA cycles 2 and 3 from junior doctors and nurses to assess effectiveness of interventions implemented. Subsequently, awareness was raised at senior doctor department meetings and physical stickers on COWs.

## Results

**Fig. 5:**
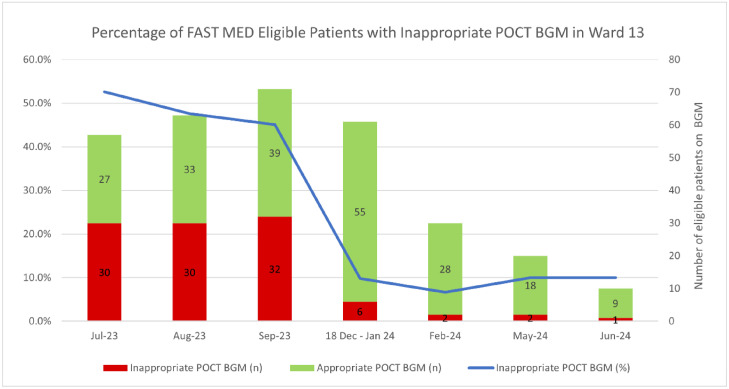
Change in inappropriate BGM from July 2023 to June 2024.

Our study team audited three times over a 7-month duration for inappropriate BGM (Fig. 5).

PDSA 1 (July–September 2023): 49.5% (*n* = 92) out of 186 patients had inappropriate BGM.

PDSA 2 (December 2023 – January 2024): 8.1% (*n* = 5) out of 136 patients had inappropriate BGM.

PDSA 3 (February–June 2024): 8.3% (*n* = 5) of 60 patients had inappropriate BGM.

Overall reduction of inappropriate BGM was from 49.5% to 9.2% over 7 months.

Qualitative feedback revealed that both junior and senior doctors brought this practice to other wards that they were subsequently rotated to. Nurses felt empowered and confident in discussing frequency of BGM with doctors.

The extrapolated cost saving is S$564,818.80 (£421,185.38) annually, based on an average number of 8,292 patients admitted to GM annually, with the assumptions that there will be a 40% reduction in patients with inappropriate BGM monitoring resulting in an average reduction of three episodes of inappropriate BGM per patient-day after the first 48 h of their admissions according to our workflow. Using approximately 1 min per BGM, 663.4 nursing hours is saved annually.

**Fig. 6:**
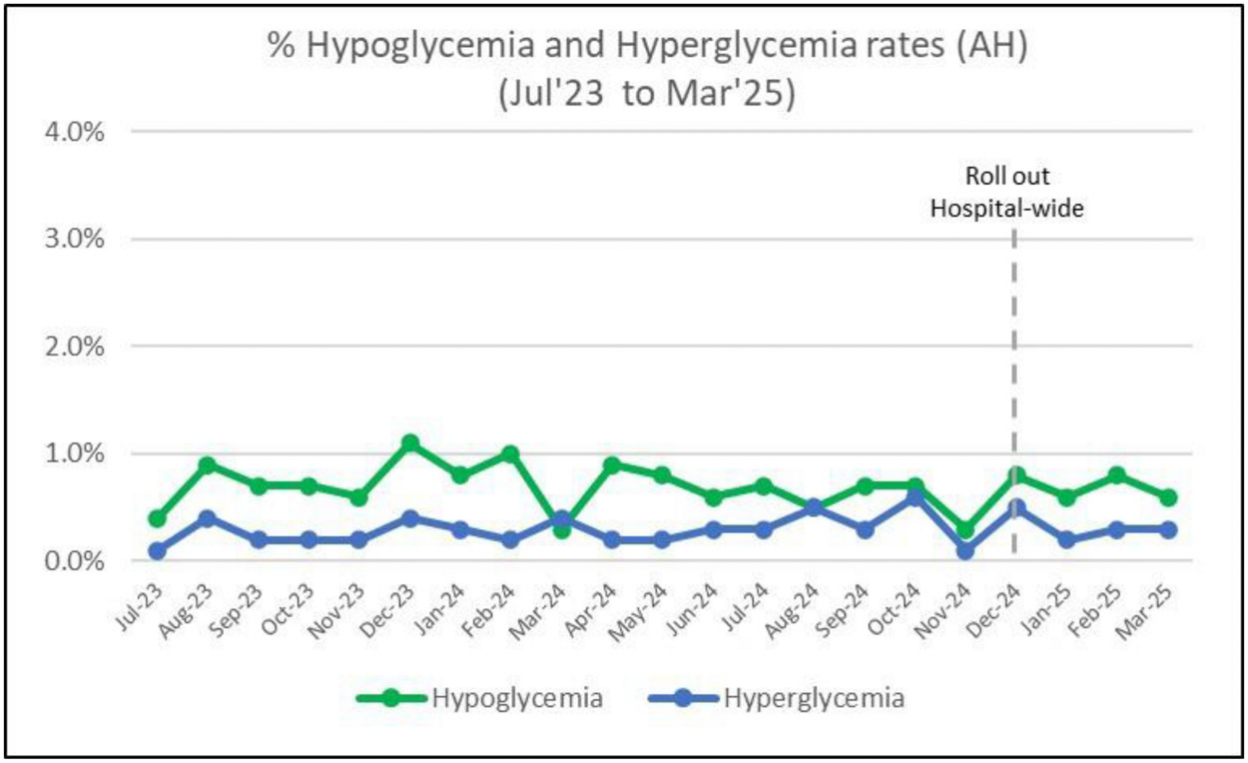
Adverse event rates of hypoglycaemia and hyperglycaemia from July 23 to March 2025.

There was no significant change in the adverse event rates of hypoglycaemia and hyperglycaemia after interventions and remained mainly below 1.0% for both outcomes over 7 months (Fig. 6). This emphasises the safety of the clinical guidelines while ensuring an acceptable range of capillary blood glucose level.

## Discussion

### Conclusions

Firstly, this quality improvement project emphasises the high prevalence of inappropriate BGM in the inpatient setting. Secondly, establishing clinical guidelines with a subspecialty department is effective and maintaining patient safety with the stable adverse event rates. Next, the project demonstrates that consistency is key to change – overcoming ingrained habits, such as defensive medicine, requires sustained commitment and robust leadership support to set high-value care by example.

Since December 2024, our project has been spread hospital-wide for all eligible patients in the medical and surgical specialties. It has been adopted by the National University Health System cluster as an Appropriate Care project and recognised with an excellence award at the cluster quality day event.

Strengths of the project are that it is easily reproducible and sustainable, with low-cost interventions that pivot on empowering individuals towards behavioural changes through consistent, structured education and collaboration between multidisciplinary healthcare teams. It has a high downstream impact for patients and the healthcare system through meaningful quality improvement. By working together, hospitals can deliver safe and cost-efficient care without compromising its quality.

Lastly, there is potential to engage artificial intelligence (AI) in this project, as AI can aid in identifying suitable patients and providing suggestion to adjust BGM monitoring frequency according to guidelines. AI could significantly reduce inefficiency in healthcare, improve patient flow and experience, and enhance patient safety.^8^

There were some limitations to our study. Firstly, the existing inclusion and exclusion criteria may render it not generalisable to a proportion of patients who come in with fluctuating changes to clinical status or higher medical complexity. There is room to explore how these criteria may be adjusted with different patient profiles and be safe in the frequency of monitoring across different subspecialties. Secondly, the project was only carried out for 7 months and the sustainability of these efforts will need to be observed over a longer period. Investigator bias could have arisen from the subjectivity of whether a test was ‘appropriate’ or not when it came to contentious clinical conditions. To minimise this, we disregarded a case from our data if clinical complexity was too high. Lastly, this project was carried out in a single hospital, and further assessment is required to determine its replicability in other hospitals with different structures.

Ultimately, physicians need to exercise their own discretion when applying these guidelines to their patients, especially for patients at risk of glycaemic variability such as malnourished patients or elderly patients with behavioural problems and erratic oral intake. Patient safety must always be prioritised while optimising healthcare resources use.

## CRediT authorship contribution statement

**Sophia Shuen Yii Ng:** Writing – original draft, Project administration, Formal analysis, Conceptualization, Data curation. **Yan Ling Lai:** Conceptualization, Data curation, Formal analysis, Project administration, Writing – review & editing. **Pei Yi Lee:** Data curation, Formal analysis, Writing – review & editing. **Karen Cera:** Conceptualization, Data curation, Writing – review & editing, Project administration. **Esther Melissa Michael:** Data curation, Writing – review & editing, Project administration, Conceptualization. **Xiao Juan Chen:** Writing – review & editing, Project administration. **Esther Huiyun Lin:** Data curation, Writing – review & editing. **Schneider Wong:** Conceptualization, Supervision, Writing – review & editing, Project administration. **Ya Yuan Nicole Chong:** Conceptualization, Writing – review & editing. **Desmond B Teo:** Writing – review & editing, Supervision, Project administration, Conceptualization.

## Declaration of competing interest

The authors declare that they have no known competing financial interests or personal relationships that could have appeared to influence the work reported in this paper.
